# The Dead Do Tell Tales: Using Pathology Data From Cetacean Necropsy Reports to Gain Insights Into Animal Health

**DOI:** 10.1002/ece3.72119

**Published:** 2025-09-10

**Authors:** Rachel L. Lennon, Jennifer Storm, Rylyn Koger, Elleigh Thompson, Rosie S. Williams, Mark P. Dagleish, Simon A. Babayan, Mariel T. I. ten Doeschate, Nicholas J. Davison, Andrew C. Brownlow

**Affiliations:** ^1^ School of Biodiversity, One Health and Veterinary Medicine University of Glasgow, University Avenue Glasgow UK; ^2^ Centre for Ecology and Conservation University of Exeter Penryn, Cornwall UK; ^3^ Zoological Society of London Institute of Zoology London UK

**Keywords:** cetacean, health, machine learning, necropsy report, pathology data

## Abstract

Assessing long‐term population health is an important feature of wildlife monitoring and is essential for understanding population resilience. Quantitative assessments of health indices are essential for effective monitoring yet they remain a challenge for cetaceans due to their highly mobile and largely cryptic nature. Quantified assessments of cetacean health typically rely on evaluating body condition, which reflects energetic reserves and nutritional status. However, this method may not capture sub‐lethal impacts or comorbidities resulting from chronic or cumulative stressors. Necropsies of stranded individuals provide unique insight into the morbidity of an individual and offer clues about the pressures an individual has faced over its lifetime, making necropsy reports a valuable source for understanding health. However, the predominantly qualitative and narrative format of necropsy reports limits their utility for systematic statistical analysis, thereby constraining their application in generating robust, population‐level inferences. Using 349 necropsy records of harbour porpoises ( *Phocoena phocoena* ) stranded on the Scottish coastline as a case study, we developed a structured pathology database suitable for statistical analysis. The application of unsupervised and supervised machine learning techniques has revealed underlying patterns in harbour porpoise pathology. This has shown that necropsy data may provide a more comprehensive insight into health than traditional methods. We identified the respiratory and hepatic systems as explaining a high degree of variation in the data. This highlights these organ systems as priority areas for data collection and subsequent analysis. Patterns in the data revealed underlying compromised health, particularly in adult harbour porpoises, suggesting a vulnerability to additional stressors in this group. Our approach demonstrates how pathology data from necropsy reports can be used to derive population‐level health insights, offering a tool for monitoring the impacts of environmental and anthropogenic pressures on wildlife populations.

## Graphical abstract

Assessing long‐term health in wildlife populations remains challenging, especially for cetaceans, whose mobility and cryptic behaviour limit traditional assessments based solely on body condition. By analysing 349 necropsy records of harbour porpoises stranded along the Scottish coastline, we created a structured pathology database and applied machine learning techniques to identify patterns in organ‐specific morbidity, highlighting the respiratory and hepatic systems as key indicators of health. Our findings reveal underlying compromised health in adult harbour porpoises, suggesting reduced resilience to additional stressors. They demonstrate that necropsy data can provide robust, population‐level insights into the impacts of environmental and anthropogenic pressures on wildlife.

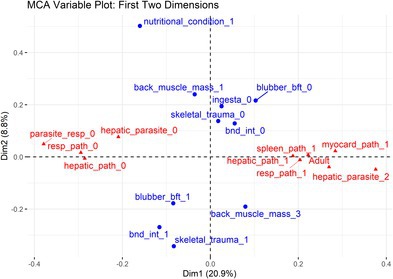

## 1 Introduction

Monitoring health is a key component of wildlife surveillance, offering valuable insights into reproductive capacity, the effects of stressors and population resilience (King et al. 2023; IJsseldijk et al. 2024). Populations with higher resilience are better equipped to adapt to changing conditions and recover from disturbances, whereas populations with compromised health are more vulnerable to declines or collapse under additional pressures (Stephen 2022). As wildlife populations are increasingly exposed to multiple, often overlapping stressors, ranging from natural (e.g., seasonal temperature fluctuations, endemic diseases, predation) to anthropogenic (e.g., climate change, pollution, biodiversity loss, bycatch and entanglement), understanding resilience is essential (Fraser and MacRae 2011; Tyack et al. 2022; Chen et al. 2024). Furthermore, the health of upper trophic‐level animals is often considered a critical indicator of ecosystem health, as these species are representative of changes in their environment (Smits and Fernie 2013). A decline in predator health can signal broader ecological imbalances, such as shifting prey availability, habitat degradation, or bioaccumulation of pollutants, with cascading effects throughout the ecosystem (Cohen et al. 2014; Williamson et al. 2021). Deteriorating wildlife health can have implications for human health, particularly through the emergence and spread of zoonotic diseases (Artois et al. 2009). Additionally, small cetaceans can act as sentinels for the bioaccumulation of persistent organic pollutants, heavy metals and harmful algal toxins, especially in prey species that overlap with those targeted by fisheries for human consumption (Lefebvre et al. 2016; Williams et al. 2020; von Hellfeld et al. 2024). Therefore, accurate and effective methods for assessing wildlife health are essential not only for assessing population resilience but also for informing the ‘One Health’ approach to optimise the health of animals, people and ecosystems that are closely linked (Gulland et al. 2025).

Cetaceans are considered sentinels of marine ecosystems as they reflect changes occurring in lower trophic levels (Williamson et al. 2021). As long‐lived, frequently wide‐ranging marine mammals, they are exposed to a complex array of stressors over prolonged time periods (Bossart 2011; Halpern et al. 2015; Nabe‐Nielsen et al. 2018). Monitoring the health of these highly mobile and often cryptic animals poses significant challenges. Traditional assessments of health in marine mammals have relied heavily on assessing body condition (BC), such as through blubber thickness, or body mass index (mass kg/length m^2^), as proxies for health (Kershaw et al. 2017). While these metrics reflect energetic reserves and nutritional status, they offer a limited view of overall health and may not capture sub‐lethal impacts or comorbidities arising from chronic or cumulative stressors, such as contaminant exposure, disease or prolonged physiological stress (Wilder et al. 2016). Furthermore, changes in BC often become apparent only after health has become severely compromised, reducing its effectiveness as an indicator of morbidity (Aguilar et al. 2007). BC also maps closely to natural variations in size that are not related to health, such as across age classes, in response to changing sea temperatures, and seasonal energetic investments related to reproduction and migration (Lockyer et al. 2003). As such, there is a growing need for more comprehensive and sensitive approaches to understanding cetacean health that can better inform how stressors impact reproductive success and survivability.

While BC remains a primary method for assessing cetacean health, alongside external indicators such as skin condition and, in live animals, emerging techniques like breath sampling, a systematic necropsy can provide a far more detailed understanding of an individual's health status prior to its death (IJsseldijk et al. 2019). Necropsies provide a critical appraisal of pathology, giving insights into proximate causes of death, organ functionality, disease presence and absence, and signs of trauma or anthropogenic interactions (Read and Murray 2000). Necropsies can also provide insight into morbidity of an individual and the historical pressures the animal has faced over its lifetime, making necropsies a valuable source of data for understanding animal health (IJsseldijk et al. 2019). However, necropsies have traditionally been recorded in prose, with observations often uncoded and inconsistently structured, limiting their utility for broader population‐level health inferences through statistical analysis. A recent study by IJsseldijk et al. (2024) demonstrated that it is possible to quantify necropsy reports and that pathology data can be used to detect patterns in causes of death. By applying this framework and statistical methodology, pathological data could be used to make health inferences, allowing for more comprehensive assessments of population vulnerability and the impacts of cumulative stressors. In doing so, necropsies can serve as a powerful tool not just for understanding individual mortality but also for wider population level health.

Here, we developed a framework for codifying proposed pathology into categorical data and used this to input findings from harbour porpoise (*Phocoena phocoena*) necropsy reports generated by the Scottish Marine Animal Stranding Scheme (SMASS). Unsupervised and supervised machine learning techniques were applied to these data and used to determine important features that explain variation in pathology, identify typical pathological profiles of harbour porpoises and explore how well these features predict BC in porpoises to assess the effectiveness of BC as a health indicator. This approach explores how data from necropsy reports can be used as a tool for understanding population health. It provides insights into population resilience and satisfies the ‘One Health’ approach.

## 2 Methods

### 2.1 Data Collection

The Scottish Marine Animal Stranding Scheme (SMASS) has conducted necropsies on harbour porpoises stranded in Scotland since 1992 (SMASS 2024). These examinations document both gross and histological findings to determine and assign a proximate cause of death while also noting any significant pathological observations (IJsseldijk et al. 2019). The term ‘cause of death’ is used here to refer to the broad classification assigned during necropsy, reflecting the likely external or biological scenario leading to death, rather than the specific physiological mechanisms that are more accurately referred to as ‘circumstance of death’ (Tomo and Kemper 2022). In this study, we included reports from 2009 to 2024, covering the last 15 years of SMASS during which the personnel conducting the examinations remained consistent.

### 2.2 Pathological Framework Development

To codify necropsy observations, a framework for quantitative pathology variables was developed. This allowed for the retrospective assessment of prose‐form necropsy reports to transform qualitative observations to quantitative variables for analytical purposes. Building on the variables identified by IJsseldijk et al. (2024), a coded framework of 38 variables was generated encompassing a variety of potential observations and diagnoses. These variables included location and environment data, standard signalment variables and pathophysiological variables that were aggregated in some cases (e.g., bronchopneumonia and verminous pneumonia are both covered by the pneumonia variable) but still retain similar underlying aetiology and chronicity. A selection of five experts working for stranding schemes in the United Kingdom and regularly conducting marine mammal necropsies were consulted to informally review the variables and were asked to provide their input on variable refinements, the levels provided for each variable and the addition or omission of variables. The final pathology framework was then used for retrospective codifying of necropsy reports (Table 1).

**TABLE 1:** Quantitative variables and their associated description used in a framework for codifying pathology data.

Name	Variable	Levels	Description	Insight	Examination
ID	mref	Character	The ID value assigned to each individual harbour porpoise	Context	Stranding
Recorder ID	recorder	1 = recorder a, 2 = recorder b, 3 = recorder c, 4 = recorder d	ID of recorder that retrospectively input necropsy report	Context	Context
Location	location	1 = West, 2 = North, 3 = East	The region the carcase was found stranded	Context	Stranding
Season	season	1 = winter (Dec, Jan, Feb), 2 = spring (Mar, Apr, May), 3 = summer (Jun, Jul, Aug), 4 = autumn (Sep, Oct, Nov)	Season of stranding by grouped months	Context	Stranding
Pathologist	pathologist	1 = pathologist 1, 2 = pathologist 2, 3 = pathologist 3, 4 = pathologist 4, 5 = pathologist 5	ID of pathologist that conducted the necropsy. All pathologist were considered specialist in marine mammal post‐mortems, with professional veterinary training and/or a minimum of 10 years' experience with marine mammal pathology	Context	Gross
Decomposition score	decomp	1 = freshly dead, 2 = mild decomposition, 3 = moderate decomposition, 4 = advanced decomposition	A score of decomposition status based on visual assessment of the carcase as outlined by IJsseldijk et al. (2019)	Context	Gross
Post‐mortem scavenger damage	pm_scavenge	0 = absent, 1 = mild, 2 = moderate, 3 = severe	A score of the degree of post‐mortem scavenger damage on the carcase prior to necropsy. Mild scavenger damage involved small areas of superficial damage not affecting anatomical integrity or key organs, moderate referred to large or deep wounds with partial loss of soft tissues but with core organs still intact, and severe was extensive tissue removal, often including exposure or loss of organs, with large parts of the carcass missing	Context	Gross
Frozen	frozen	0 = no, 1 = yes	Indication of whether the carcase was frozen prior to conducting the necropsy	Context	Gross
Sex	sex	0 = female, 1 = male	Sex of animal	Context	Gross
Age class	age	1 = neonate, 2 = juvenile, 3 = adult	Age class based on total body length, where animals < 90 cm were categorised as neonates, those between 91 and 130 cm were categorised as juveniles and those > 130 cm were considered adults (Lockyer et al. 2003)	Context	Gross
Cause of death category	cfod	1 = blunt‐force trauma, 2 = bottlenose dolphin attack, 3 = bycatch, 4 = grey seal predation, 5 = infectious disease, 6 = live stranding, 7 = neonatal death, 8 = other/undetermined, 9 = starvation/emaciation	Proximate cause of death assigned to case by attending pathologist	Context	Gross
Back muscle mass	back_muscle_mass	1 = concave, 2 = flat, 3 = convex	The outlining of the animal's back muscles on cranial perspective	Energetics	Gross
Nutritional condition	nutritional_condition	1 = poor, 2 = moderate, 3 = good, 4 = very good	Visual assessment of musculature and blubber thickness	Energetics	Gross
Ingesta	ingesta	0 = absent, 1 = recent, 2 = past	Prey items in the stomach, with recent being fleshy undigested prey and past being hard tissues (e.g., otoliths, squid beaks, fisheyes)	Energetics	Gross
Natural ingesta	ingesta_natural	0 = no, 1 = yes	Environmental non‐prey material in the stomach including plants, stones, wood, seaweed, mud	Energetics	Gross
Hepatic jaundice	hepatic_jaundice	0 = no, 1 = yes	Yellow colour to the liver	Energetics	Gross, histology
Hepatic lipidosis	hepatic_lipidosis	0 = no, 1 = yes	Fatty change characterised by vacuolation of hepatocytes	Energetics	Histology
Live stranding	live_stranding	0 = no, 1 = yes	Observed live stranding of animal	Context	Stranding, gross
Seal interactions	seal_int	0 = no, 1 = yes	Puncture or bite lesions consistent with seal interactions	Trauma	Gross
Bottlenose dolphin interactions	bnd_int	0 = no, 1 = yes	Rake marks consistent with bottlenose dolphin teeth distance (11–13 mm)	Trauma	Gross
Fisheries interactions	fisheries_int	0 = no, 1 = yes	Lesions consistent with fisheries interactions including encircling lesions, or any other lesions that may indicate entanglement in rope or netting	Trauma	Gross
Skin lesions	skin_lesion	0 = absent, 1 = mild, 2 = moderate, 3 = severe	Active wound on the skin, of viral, bacterial, fungal, or unknown aetiology	Infection	Gross, histology
Blunt force trauma	blubber_bft	0 = no, 1 = yes	Bruising in the blubber or subcutaneous muscle indicative of blunt force trauma	Trauma	Gross
Skeletal trauma	skeletal_trauma	0 = absent, 1 = acute, 2 = healed	Presence of skeletal fractures. Acute fractures having evidence of occurring antemortem (e.g., associated with haemorrhage and bruising)	Trauma	Gross
Central nervous system pathology	cns_path	0 = no, 1 = yes	Meningitis, encephalitis, meningoencephalitis, including signs of inflammation or infiltration of inflammatory cells	Infection	Histology
Myocardial pathology	myocard_path	0 = no, 1 = yes	Myo‐ or pericarditis, and/or signs of inflammation or infiltration of inflammatory cells	Infection	Gross, histology
Oedema in the respiratory system	oedema_resp	0 = no, 1 = yes	Stable foam and/or free fluid in the trachea and/or interstitial pulmonary oedema. Pulmonary congestion	Trauma	Gross, histology
Parasites in the respiratory system	parasite_resp	0 = absent, 1 = mild, 2 = moderate, 3 = severe	Parasites in the conducting airways and/or pulmonary vessels	Infection	Gross
Respiratory pathology	resp_path	0 = no, 1 = yes	Pneumonia (broncho, verminous or other) and/or emphysema, including signs of inflammation or infiltration of inflammatory cells	Infection	Histology
Parasites in the middle ear and sinuses	parasite_ear	0 = absent, 1 = mild, 2 = moderate, 3 = severe	Parasites in the peri‐bullae sinuses and/or tympanic cavity	Infection	Gross
Alimentary pathology	ailmentary_path	0 = no, 1 = yes	Lesions in the oral cavity and/or oesophagus and/or small and large intestines. Including gastritis, oral ulcers, gastric ulcers, oesophagus ulcers and gastritis	Infection	Gross, histology
Parasites in the gastrointestinal tract	parasite_git	0 = absent, 1 = mild, 2 = moderate, 3 = severe	Nematodes, cestodes, trematodes in stomach lumen, stomach wall and/or intestine	Infection	Gross
Hepatic pathology	hepatic_path	0 = no, 1 = yes	Hepatitis and/or cholangitis. Including signs of inflammation or infiltration of inflammatory cells	Infection	Histology
Hepatic parasites	hepatic_parasite	0 = absent, 1 = mild, 2 = moderate, 3 = severe	Trematodes in hepatic ducts	Infection	Gross
Reproductive pathology	repro_path	0 = no, 1 = yes	Including mastitis, endometritis, orchitis, severe obstructions e.g., mineralisation causing blockages, dystocia, inflammation	Infection	Gross, histology
Adrenal pathology	cmr_adrenals	0 = no, 1 = yes	A ratio of the cortex to medulla in the adrenals that is greater than 2:1 or described as inflamed and/or enlarged	Energetics	Gross, histology
Spleen pathology	spleen_path	0 = no, 1 = yes	Including inflammation or infiltration of inflammatory cells	Infection	Gross, histology
Lymph node pathology	lymph_path	0 = no, 1 = yes	Enlargement of any lymph node	Infection	Gross, histology

### 2.3 Retrospective Assessment of Necropsy Reports

For this study, necropsy reports from 2009 to 2024 covering 349 cases were used. Four recorders retrospectively codified the necropsy reports following the developed pathological framework. Each case had a complete necropsy report and, where applicable, a histology report.

Calibration of assessment between recorders was conducted prior to the commencement of codification. This involved all four recorders assessing the same report and comparing findings to ensure that there was consistency of data input. Consistency of assessment of similar words was also solidified to ensure that all recorders interpreted prose in a similar way. Once consistency was achieved, true retrospective analysis began. The reports were split randomly and evenly among all four recorders. The assessments were all conducted simultaneously, allowing for immediate discussions and checking of consistency between recorders.

### 2.4 Recorder Bias Validation and Correction

Following an initial round of data input, recorder bias was evaluated using 100 quantified cases. A loop of generalised linear models (GLM) was carried out with *recorder* as a fixed effect, and each of the remaining 37 variables as the response variable, using RStudio and R version 4.2.2 (R Core Team 2022). The models were fitted with a binomial distribution family and a link‐logit function. To account for the increased chance of type I errors from multiple hypothesis testing, the typical *p*‐value threshold of 0.05 was adjusted following Bonferroni correction (adjusted α = 0.001) (University of Berkeley, n.d.). At this threshold, recorder effects remained significant for two variables: myocardial pathology and spleen pathology.

To mitigate identified bias, a single individual reviewed all necropsy reports, correcting minor transcription errors and harmonising inconsistencies between recorders. A second round of GLMs was then conducted on the revised dataset to test for residual recorder bias. At the Bonferroni‐adjusted threshold, no pathology variables were significant for recorder. Therefore, levels of recorder bias were deemed acceptable to carry out the analysis on the second checked data.

### 2.5 Missing Data

A total of 349 necropsy reports were codified throughout this study, but only complete cases with values for all variables were included in the final analysis. Of the 349 cases, only 164 met these criteria and were retained for further analysis. It is important to establish the drivers of incomplete necropsy reports so that limitations of data collection can be understood. To determine which variables best predict missing data, a generalised linear model was run on a binary variable of complete (1) or incomplete (0) necropsy report, as a function of decomposition (freshly dead = 97, mild decomposition = 137, moderate decomposition = 74, advance decomposition = 10), post mortem scavenger damage (absent = 200, mild = 37, moderate = 55, severe = 26) and frozen (no = 178, yes = 140) as factor variables, with a binomial error distribution family. Odds ratios were derived by back‐transforming the model outputs.

### 2.6 Data Analysis

Extensive data exploration and visualisation were performed prior to data analysis to assess trends in the data. Unsupervised machine learning techniques were utilised to explore the structure within the data and determine clustering of pathological variables (Alloghani et al. 2020). Supervised machine learning was used to ascertain if pathological variables can be used to predict BC, a traditional proxy of health.

#### 2.6.1 Cramer's V

Assessing the correlation between pathological variables indicates which features tend to co‐occur, helping to elucidate underlying relationships and recurring patterns that may reflect common morbidities and mortalities (Akoglu 2018; IJsseldijk et al. 2024). Cramer's V correlation coefficient allows for the calculation of correlation between categorical variables. It is a measure of the effect size of a chi‐squared test of independence, producing a value between 0 and 1 with 1 being complete association and 0 being no association (Akoglu 2018).

Here, the correlation between all pathological variables was assessed using Cramer's V correlation coefficient with the DescTools and vcd packages in R (Signorell 2017; Meyer et al. 2024). A correlation matrix calculating the Cramer's V coefficient between all variables was generated. The correlation matrix was visualised using the corrplot package to illustrate all associations (Wei and Simko 2024). Any pair of variables with a coefficient that was greater than 0.5 was assessed visually using barplots with the ggplot2 package to determine the directionality of the observed association (Wickham 2016).

A second matrix was generated to illustrate the percentage presence of pathological features for each cause of death type to visualise the rate of occurrence and how this differs across the causes of death. Variables with multiple levels were transformed to presence/absence where possible to allow for inclusion. For each type of cause of death, the percentage of cases that had each of the retained pathological variables present was calculated. A matrix was then generated using ggplot2 to illustrate these values.

#### 2.6.2 Multiple Correspondence Analysis

Establishing patterns in pathology is key for understanding overall health, while determining which variables and organ systems are most important for driving this variation can inform and refine data collection and analysis efforts. The pathological variables included in the analysis encompass both gross and histological findings, which may partially overlap due to the biological co‐occurrence of parasitic infections and associated tissue changes. As a result, we chose to employ multivariate methods for their ability to handle correlated categorical data and identify meaningful patterns of co‐occurrence. Consequently, rather than treating each variable as independent, the analysis acknowledges and leverages these relationships.

Multiple correspondence analysis (MCA) can be used on multiple categorical variables to collapse datapoints into multidimensional Euclidean space and assess underlying structures in data (Broeksema et al. 2013). This approach enables the identification of key variables that explain variation in the data, reveals how these variables and their levels relate to one another and highlights associations between variables based on similar individual profiles.

An MCA with a Burt table was conducted on a dataset excluding the cause of death variable to blind test for patterns unrelated to proximate cause of death, using the FactoMineR package (Lê et al. 2008). Eigenvalues were extracted to ascertain how well each dimension explains the inertia in the data. Eigenvalues were plotted on scree plots using the factoextra package in R (Kassambara and Mundt 2020). The ‘elbow rule’, which identifies when adding more dimensions does not improve the model further, was used to determine the optimal number of dimensions for explaining the variance in the data. The correlation ratio (*R* ^2^) values were extracted to quantify the strength of association between each variable and the 1st and 2nd dimensions, with higher values indicating that the variable contributed strongly to the inertia of that dimension. Contribution (contrib) and squared cosine (cos2) scores were then used to determine how much each level of the important variables was contributing to defining dimensions 1 and 2. The test values were also extracted to indicate positive and negative associations of variable levels with the dimensions. MCA factor maps were generated using the ten variables with the highest contribution scores to illustrate the coordinate position of variables and individuals in the first two dimensions, illustrating the relationships and associations within the established Euclidean space.

#### 2.6.3 Hierarchical Clustering

Identifying how individuals cluster within the dataset, and exploring the variables characterising each cluster, can help establish key pathological profiles. Hierarchical clustering classifies datapoints that are positioned close together based on their position within multidimensional Euclidean space (i.e., proportional variances between variables) to agglomerate datapoints into clusters within the dataset (El‐Araby et al. 2023).

The coordinates of datapoints were taken from the first dimension of the MCA and used for hierarchical classification to visualise variables that cluster together within the dataset and are thus correlated, using the FactoMineR package. A dendrogram was generated to visualise the relationship between established clusters and determine the optimal number of clusters from the data. Clusters were mapped and coloured according to levels of cause of death in order to determine how these clusters differentiate between types of cause of death.

#### 2.6.4 Random Forest Classification

Identifying which pathological variables are captured by traditional BC measures provides insight into how accurately this metric reflects the underlying pathology. A random forest (RF) regression model conducts a regression tree analysis to predict a continuous target variable (Segal 2004). The algorithm constructs multiple decision trees and determines where nodes should be placed on each tree using a random subset of data. The predictions are aggregated to provide more accurate and stable estimations. Using the randomForest package, an RF regression was conducted using body mass index (mass/length^2^) as a BC index (Kershaw et al. 2017) for each individual to determine the variables that are important for predicting this classic proxy of health (Liaw and Wiener 2002).

The dataset was randomly split into a training set (70% of the data) and a test set (the remaining 30% of the data) in order to validate the algorithm. Using a tuning grid, the best parameter values were determined for the number of trees used in the RF (ntree) with variables ranging from 500 to 1000 with step sizes of 100 and the number of variables randomly chosen at each split (mtry) with test values ranging from 2 to 10. The best RF model was chosen by evaluating its performance and predictive ability through assessing Root Mean Square Error (RMSE) and *R* ^2^ values.

The variables that were most important in predicting the classification were assessed by estimating the percentage increase in RMSE, indicating how much the model's predictive error increases when the values of a variable are randomly shuffled (Segal 2004). A higher value indicates that the variable is important for predictive accuracy in the model. The increased node purity was also assessed, which is a measure of the improvement in node purity contributed by the variable, measured using the residual sum of squares. A higher value indicates that the variable is influential for forming the structure of the RF trees.

## 3 Results

### 3.1 Drivers of Incomplete Necropsy Reports

The driving variables for incomplete necropsy reports were identified through our analysis, allowing us to understand limitations to complete data collection. The GLM test showed post mortem scavenger damage *severe* (SE ±0.56, *z* = 3.37, *p* < 0.05) and frozen *yes* (SE ±0.25, *z* = 4.28, *p* < 0.05) to significantly predict whether the necropsy report was complete. The odds of an incomplete necropsy report were 6.54 times higher (95% CI: 2.33–21.54) if the carcase had severe post mortem scavenger damage, and 2.93 times higher (95% CI: 1.80–4.83) when the carcase was frozen prior to necropsy.

### 3.2 Correlation and Co‐Occurrence of Pathology Variables

**FIGURE 1:**
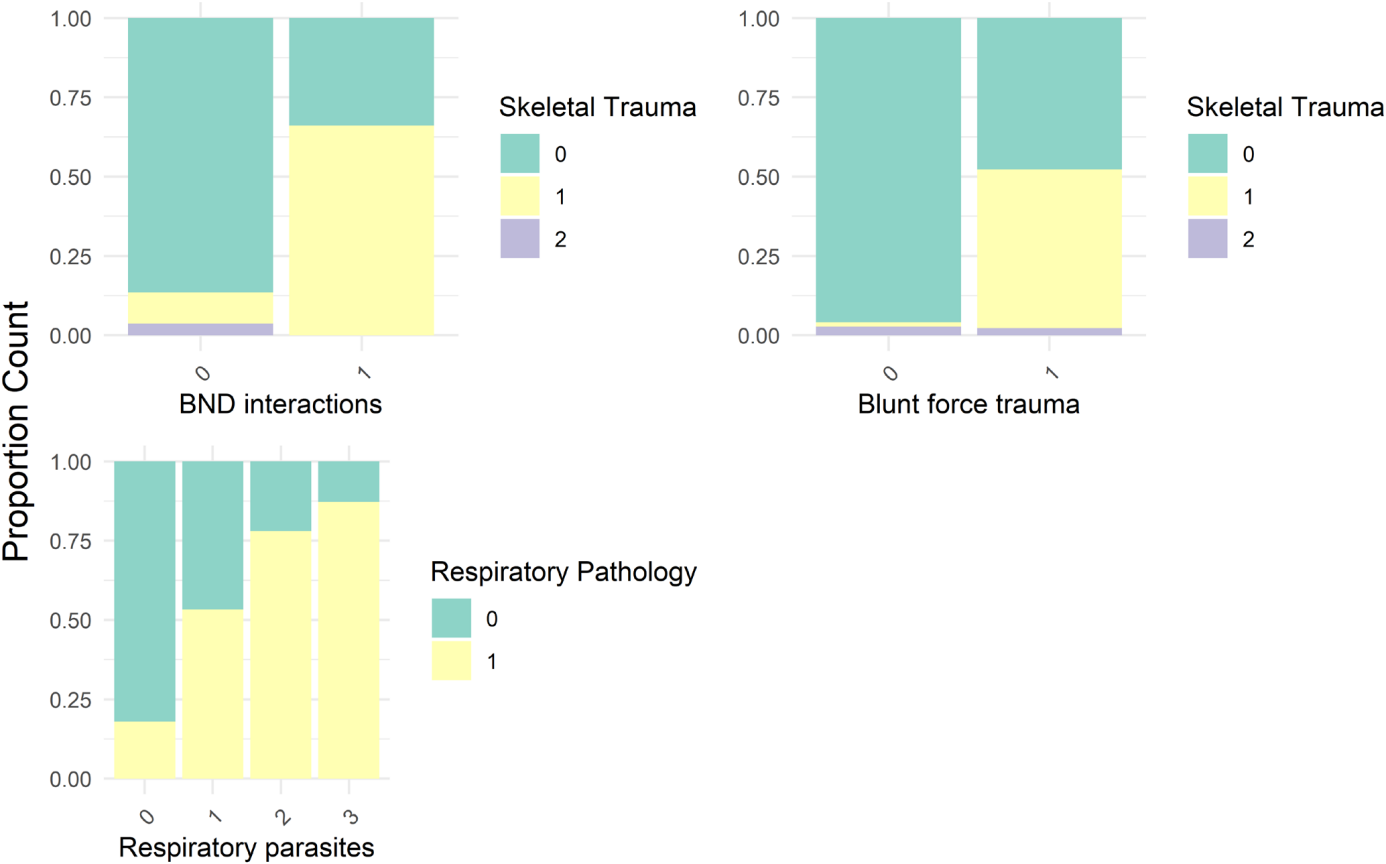
Proportional associations between variables with Cramer's V correlation value of > 0.5. Illustrated as proportional count of occurrence across total number of cases. Bottlenose dolphin (BND, *Tursiops truncatus* ) interactions, skeletal trauma, respiratory pathology, respiratory parasites and blunt force trauma are further described in Table 1.

Our analysis revealed underlying correlations and co‐occurrences between pathology variables. There were 60 combinations of variables that had a Cramer's V correlation coefficient of > 0.25, indicating a strong association (Figure S1). Combinations with Cramer's V values exceeding 0.5 were bottlenose dolphin interactions and skeletal trauma; blunt force trauma and skeletal trauma; and respiratory parasites and respiratory pathology. Bar plots illustrating the direction of these relationships show presence is correlated with presence across the variables, following a Likert scale for respiratory pathology, which is a 4‐level variable (Figure 1). The skeletal trauma associations are between the *absence* and *acute (1)* value, with no trend seen across the *healed (2)*.

**FIGURE 2:**
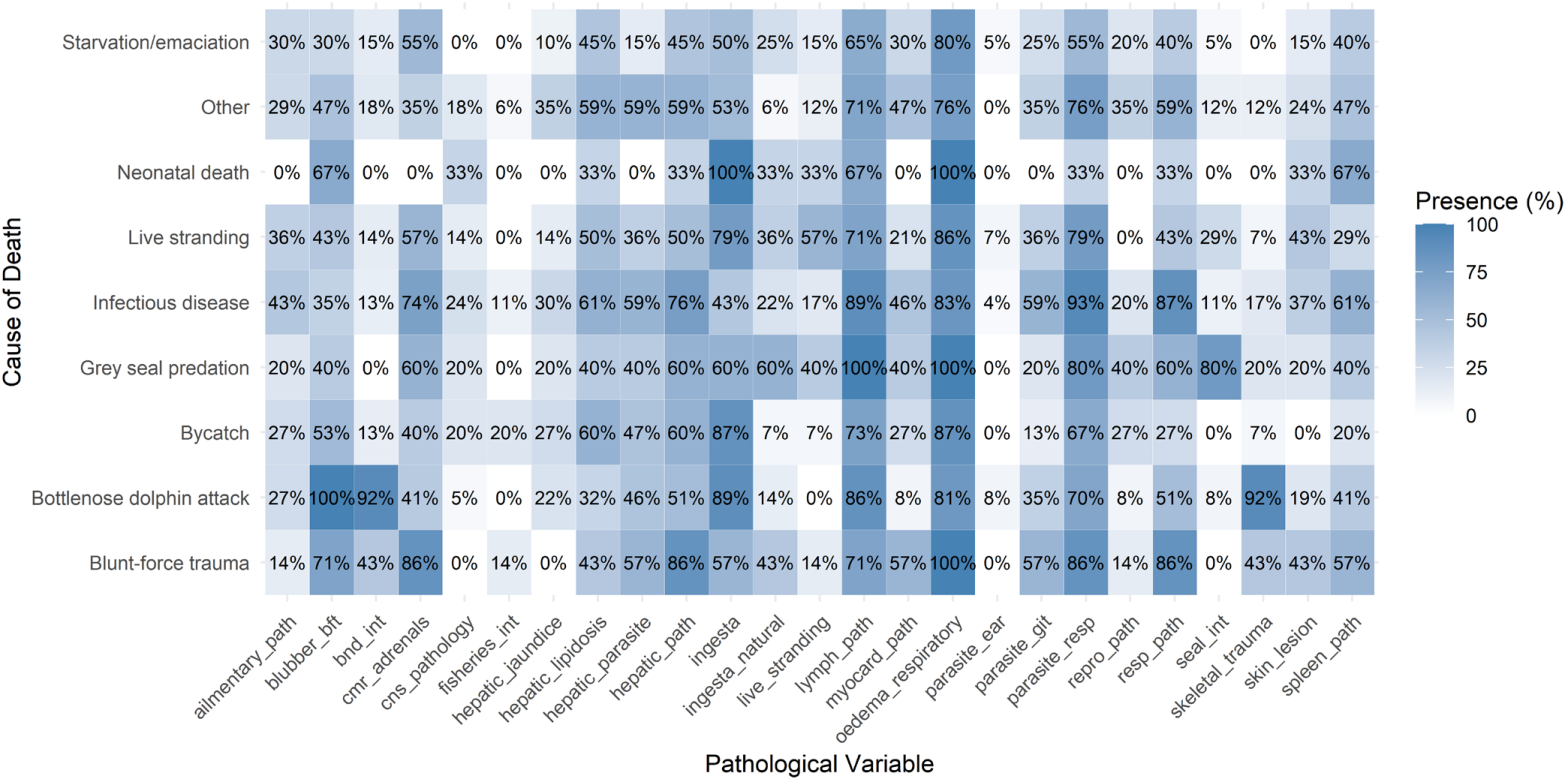
Matrix of the percentage of presence for binary pathological variables across each cause of death type. Each square is coloured by percentage, with percentage value listed for each combination. Variables are described in Table 1.

The percentage of occurrence of each pathological variable across the levels of the cause of death illustrated co‐occurrence of multiple variables (Figure 2). There were several variables that showed 100% presence for specific cause of death types, including the *presence* of ingesta and respiratory oedema for *neonatal death*, the *presence* of lymph node pathology and respiratory oedema for *grey seal (Halichoerus grypus) predation*, the *presence* of blunt force trauma in *bottlenose dolphin attack* and the *presence* of respiratory oedema for *blunt force trauma* cases.

### 3.3 Multidimensional Variation

MCA of categorical variables identified two organ systems that explain the most variation in pathological data: the respiratory system and the hepatic system. Inspection of scree plots illustrates that the most optimal number of dimensions is 4, which accounts for 42.8% of the inertia in the 64‐dimensional Euclidean space (Figure S2).

**FIGURE 3:**
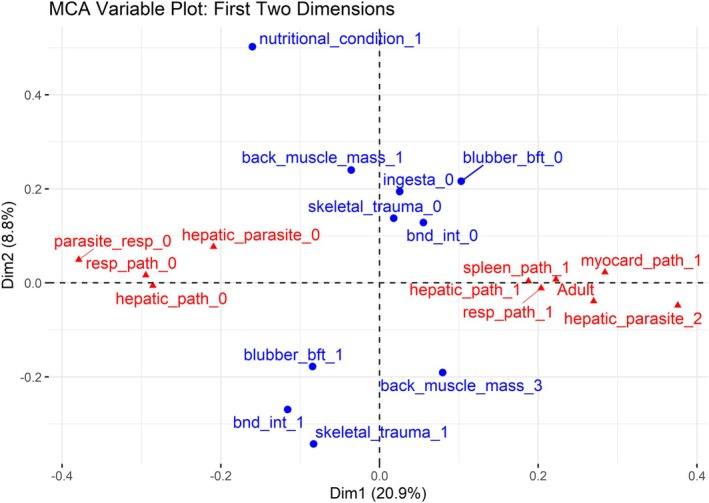
Factor plot of the first two dimensions of the Multiple Correspondence Analysis. Variables in red are the top 10 variables that contribute to dimension 1, that is they had the highest contribution and cos2 scores. Variables in blue are the top 10 variables that contributed to dimension 2. Variable levels described in Table 1.

The variables explaining most of the variation in the first dimension are respiratory parasites (*R* ^2^ = 0.47), age class (*R* ^2^ = 0.46), hepatic parasites (*R* ^2^ = 0.46), respiratory pathology (*R* ^2^ = 0.45) and hepatic pathology (*R* ^2^ = 0.40). Within these, the levels that contributed the most to the first dimension (high contribution and cos2 score) with a negative association (test values are negative) were *absent* in respiratory parasites, hepatic parasites, respiratory pathology and hepatic pathology. The levels that contributed the most to the first dimension with positive associations (test values are positive) were *adult* in the age class, *moderate* in hepatic parasites and *present* in respiratory pathology and hepatic pathology (Figure 3).

The variables explaining most of the variation in the second dimension are skeletal trauma (*R* ^2^ = 0.48), blunt force trauma (*R* ^2^ = 0.46), nutritional condition (*R* ^2^ = 0.40), bottlenose dolphin interactions (*R* ^2^ = 0.36) and back muscle mass (*R* ^2^ = 0.30). Within these, the levels that contributed the most to the second dimension and had negative associations are *absent* in skeletal trauma, back muscle mass and bottlenose dolphin interactions; *very thin* in nutritional condition and *concave* in back muscle mass. The levels that contributed the most to the second dimension with positive associations were *present* in skeletal trauma, blunt force trauma and bottlenose dolphin interactions, and *convex* in back muscle mass (Figure 3).

### 3.4 Hierarchical Clustering

Using the coordinates from the first dimension of the MCA, hierarchical clustering identified typical pathological profiles seen in stranded harbour porpoises in Scotland (Table S1). The dendrogram illustrated three key clusters in the data (Figure S3).

The first cluster was the largest and predominantly made up of *juveniles* in *good* nutritional condition, with *present* for blunt force trauma and *absent* in parasite and pathology variables. This cluster was dominated by *bottlenose dolphin attack* cause of death diagnoses. The second cluster was mainly *juveniles* and *adults* in *moderate* nutritional condition with *present* for hepatic and respiratory pathology variables, and *moderate* or *severe* for hepatic and respiratory parasite variables. This cluster was dominated by *infectious disease* cases, but also had a high number of *starvation/emaciation* cases. The third cluster was predominantly *adult* harbour porpoises in *good* nutritional condition, with *present* for hepatic and respiratory pathology variables and *moderate* for hepatic and respiratory parasite variables. This final cluster was also dominated by *bottlenose dolphin attack* cases but there was also a high number of *infectious disease* cases.

**FIGURE 4:**
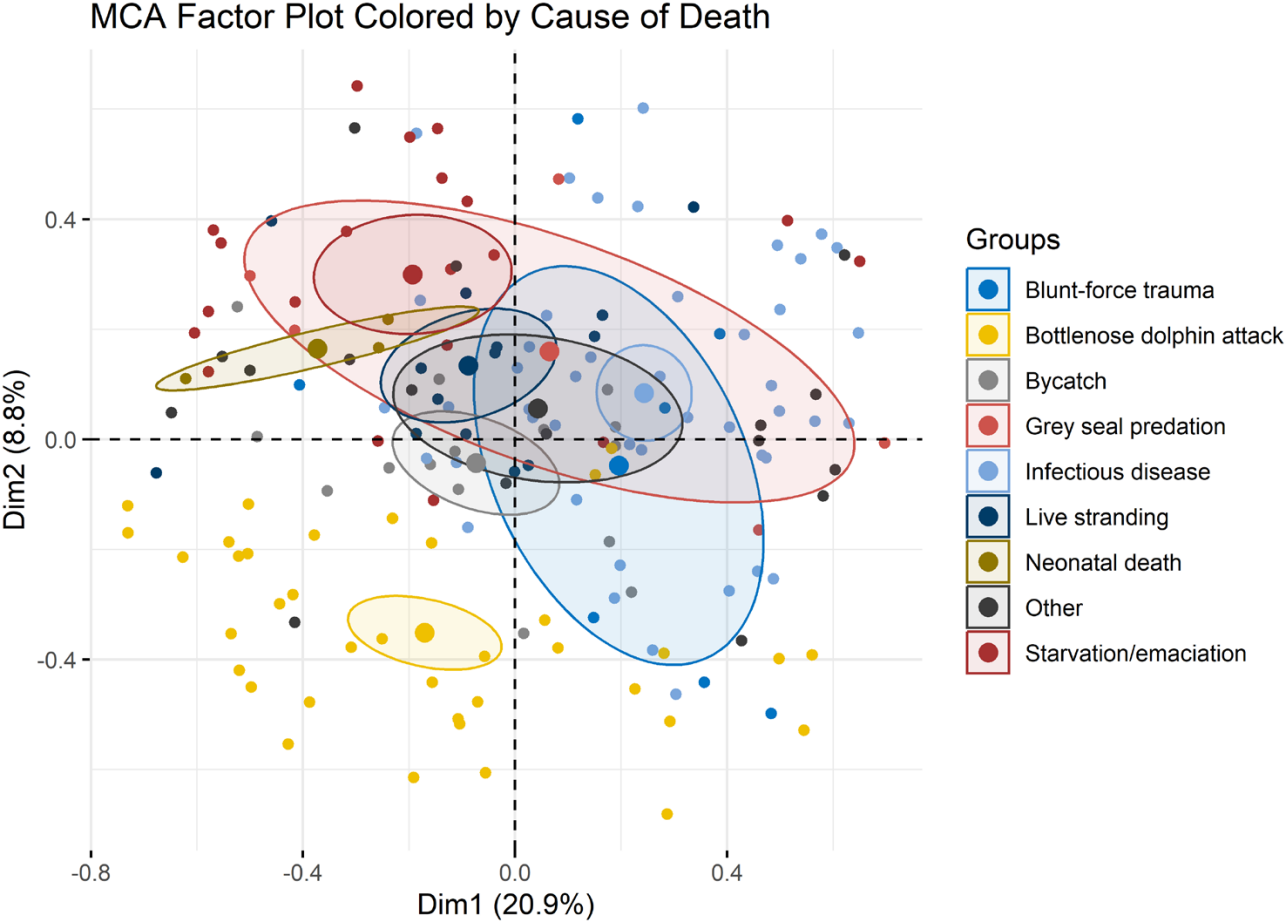
Multiple correspondence analysis factor map, with individuals coloured by their assigned cause of death. Confidence ellipses are drawn with a 0.95% confidence level for each cause of death level. Ellipses are overlapping, indicating that there is no clear distinction between cause of death types, except for the bottlenose dolphin attack.

When individuals on the MCA factor map were colour‐coded by cause of death, there was a high degree of overlap between the ellipses, except in cases of *bottlenose dolphin attacks*, which were distinct (Figure 4).

### 3.5 Random Forest Prediction of Body Condition

**FIGURE 5:**
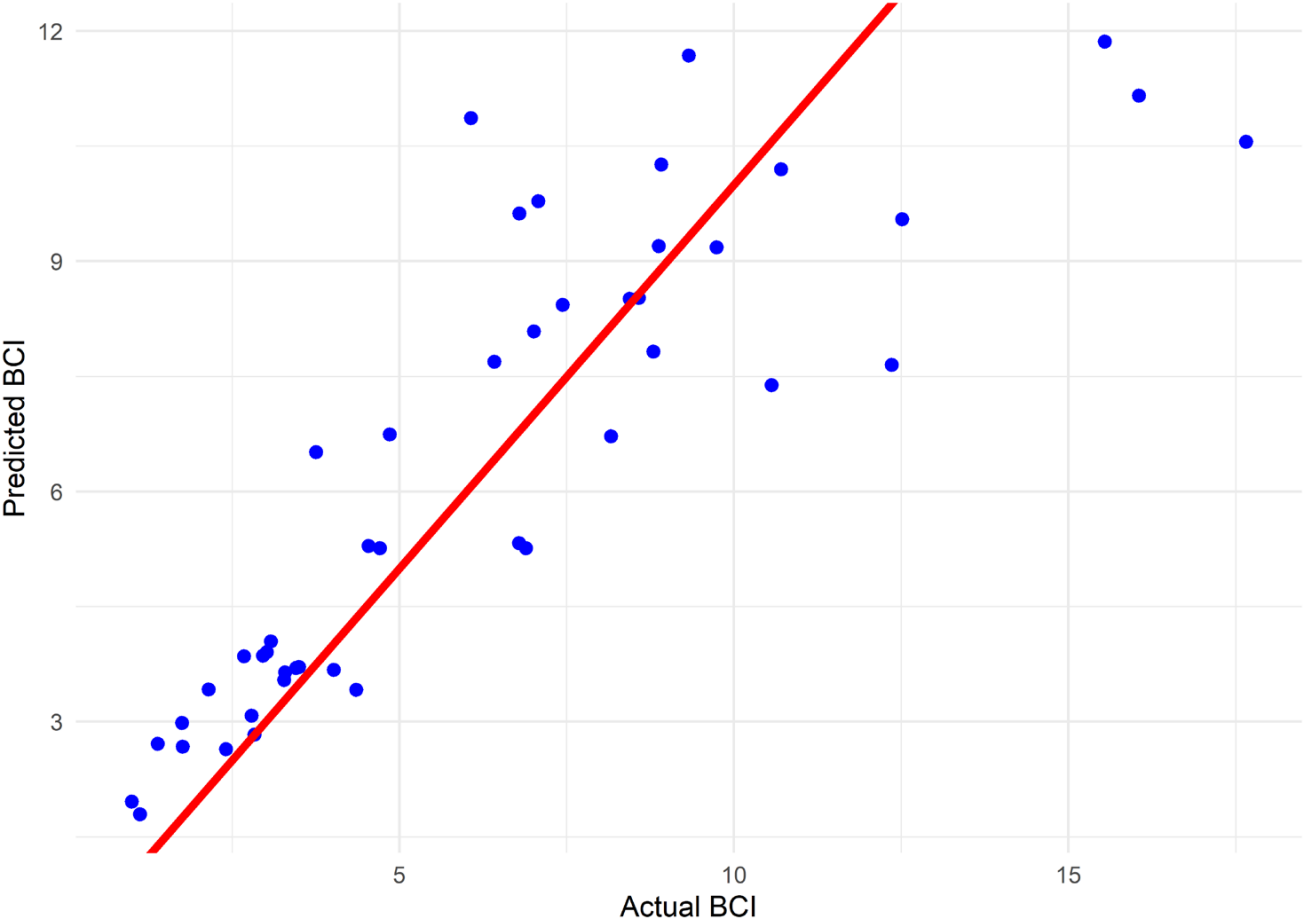
Scatterplot of the actual Body Condition Indices (BCI, weight/length^2^) of each harbour porpoise in blue point data, with a red line of the predicted BCI variables from the Random Forest regression model.

A RF classification model was able to effectively predict BC indices of harbour porpoises using pathology variables. The RF model that had the highest accuracy in predicting BC included 600 trees and mtry 10. This model had an RMSE score of 2.14, which, given the range of the BC variable (0.73–17.65), is considered relatively low and thus a good accuracy score. Different ntree values (500–1000) were tested, with the RMSE score ranging between 2.14 and 2.37, meaning the accuracy of the model did not change much with different ntree values. The *R* ^2^ of the model was 0.73, meaning 73% of the variation in BCI was captured by the pathology variables in this RF model (Figure 5).

**FIGURE 6:**
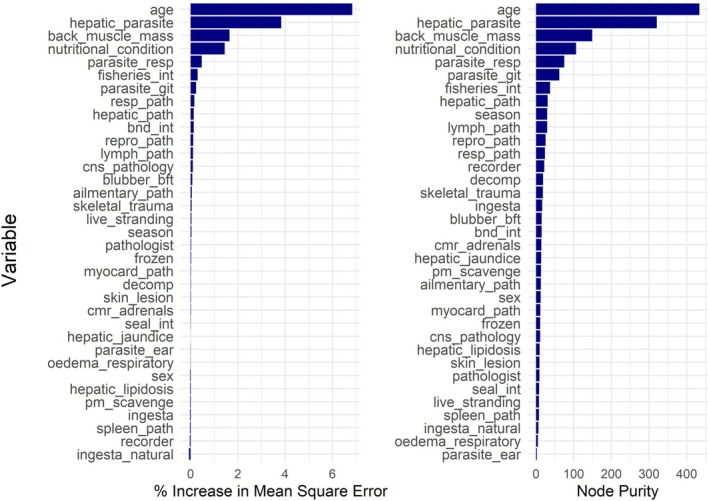
Variables used in the Random Forest classification model and how important each variable is based on the percentage increase in mean square error and increase in the purity of the node in the tree when that variable is removed from the model. Variable levels are explained in Table 1.

The variables that were most important for the predictive ability of the model as well as for formulating the structure of the trees and increasing node purity were age class, hepatic parasites, back muscle mass, nutritional condition and respiratory parasites (Figure 6). Respiratory pathology and hepatic pathology caused a < 0.5% increase in mean square error, indicating they had minimal influence on the model's predictions. The variables that were least influential to the structure of the trees were ear parasites, pulmonary oedema, natural ingesta, spleen pathology and live stranding.

## 4 Discussion

Evaluating health provides insight into the pressures faced by wildlife populations and how these pressures affect morbidity, offering a broad‐scale understanding of resilience (King et al. 2023). The findings of this study reveal a complex interplay of factors that define health in marine mammals and offer a potential shift in how populations are monitored. By quantifying pathological observations, we can uncover biological signatures that are not captured by conventional metrics. Lesions in the respiratory and hepatic systems emerge as key indicators of health status. This likely reflects the important functional roles of these organs and their vulnerability to stressors. For example, the liver plays a key role in energy metabolism and detoxification and is known to accumulate environmental contaminants (Sonne et al. 2010). The respiratory system is particularly important for diving mammals like the harbour porpoise but is easily exposed to airborne pathogens and susceptible to parasites (Jepson et al. 2000). These pathological patterns, shaped by age and showing unexpected consistency across diverse causes of death, represent a significant advancement in assessing health compared to traditional body condition indices (BCI). This systematic approach enables high‐resolution insights into individual harbour porpoise health trajectories and population‐level vulnerability to additional stressors, with broad relevance to conservation biology in the face of escalating anthropogenic pressures on marine ecosystems.

### 4.1 Important Pathological Features

We identified several key features that were important in explaining the variation in pathological data. These included respiratory pathology, respiratory parasitism, hepatic pathology and hepatic parasitism, all of which clustered together on the MCA factor plot, indicating that they co‐occur with one another. Similarly, strong associations were found in the Cramer's V analysis between the presence of parasites in an organ system and the occurrence of pathology in that same system. This association is likely due to the structural disturbance and associated immunological response caused by the presence of the parasites, which also makes the system vulnerable to secondary bacterial infections (Reckendorf et al. 2021). Pathology in the respiratory tract and its association with parasites was identified as an important variable by IJsseldijk et al., indicating the significance this feature holds across populations (2024). Indeed, pneumonia, particularly verminous pneumonia, is reported as one of the most common findings across numerous populations of porpoises (Siebert et al. 2020). Cholangiohepatitis is also reported as a common finding in porpoises and is typically believed to develop in response to a high presence of fluke parasites in the liver (Neimanis et al. 2022). However, it is difficult to determine whether parasitic infections initiate tissue damage or are a secondary consequence of pre‐existing pathology (Stimmelmayr et al. 2021). The appearance of lesions at necropsy can vary depending on the progression of the disease. Further histopathological studies would be needed to clarify the temporal sequence and prominence of these processes.

Regardless of chronology, the findings of this study highlight the respiratory and hepatic systems as important for harbour porpoise health and should be prioritised by pathologists when conducting necropsies in resource‐limited conditions, as well as for subsequent statistical analyses. The high incidence of pathology and parasitic lesions in these organs may reflect a combination of factors, including species‐specific vulnerability, regional infection pressures, nutritional status, and physiological stress, all of which can influence host susceptibility and parasite load (Pool et al. 2021). While our study did not aim to disentangle these drivers, recognising their potential contribution is important when interpreting pathological trends and setting priorities for future health surveillance.

Chronic stress may also play a role here, although detecting it can be more challenging as the associated histological changes are often subtle, variable, or dependent on the individual's physiological adaptation (Clark et al. 2006; Duijne 2012). In our study, changes in the spleen and adrenal glands were observed in some cases; however, they were not identified as key contributors in the statistical analyses, likely due to their lower frequency in the population compared to hepatic or respiratory changes. This suggests that chronic stress responses may be pathologically relevant, but they may be underrepresented in the population and thus undervalued in large‐scale analyses. This could potentially lead to an underestimation of the role that chronic stress plays in overall health and susceptibility to other disease processes. Future analyses of pathological variables could benefit from applying weights to specific findings of known relevance, even if they are infrequent, as demonstrated by Barreto et al. (2025). This approach may help ensure that biologically meaningful but rare conditions, such as those associated with chronic stress, are not overlooked in population‐level assessments.

### 4.2 Age Class‐Dependent Patterns

Age class was also highlighted as an important factor explaining the variation in data and displayed strong associations with parasite and pathology variables. The *adult* age class clustered closely with *present* for pathological variables and was more commonly associated with *moderate* for parasitic variables. This combination of variables showed strong associations in the Cramer's V analysis, illustrating that older harbour porpoises are more likely to have multiple pathologies and parasites across organ systems. This is likely a factor of higher parasite burdens in older animals due to prolonged exposure to parasites throughout their lifetime, which in turn increases the number of lesions and comorbidities in affected organ systems (Lehnert et al. 2014). Neonates were almost exclusively devoid of parasites or pathological variables, which is likely in part due to the short duration of this age class and thus limited environmental exposure. In contrast, juveniles showed high variation (*absent* to *severe*) for these features, which may reflect increasing exposure to parasite intermediate hosts, such as haddock and whiting, as they transition to independent foraging (Lehnert et al. 2014). This novel and random exposure to infectious intermediate stages of parasites likely explains the variability seen in this age class. The findings highlight a clear pattern of increasing parasite burden and risk of pathology presence with age, likely driven by cumulative exposure over time. This underscores the role of age class as a critical factor in disease susceptibility, with juveniles representing a transitional stage of variable risk.

### 4.3 Cause of Death Profiles in Harbour Porpoise

Acute physical trauma cases provide an opportunity to assess the health of harbour porpoises that have not died from chronic, debilitating conditions and thus represent the ‘general’ condition of the population. *Bottlenose dolphin attack* cases may be the most useful acute physical trauma sub‐group to observe for morbidity insights as confidence ellipses on the MCA factor plot highlight it as the only cause of death with a distinct pathological profile. Cases of *bottlenose dolphin attacks* sometimes share variables with chronic causes of death, as seen by a 51% prevalence of both respiratory and hepatic pathology. However, all cases will show the *presence* of blunt force trauma, and most will have bottlenose dolphin rake marks and *acute* skeletal trauma. This is unsurprising and matches the typical features of bottlenose dolphin kills seen in the literature (Ross and Wilson 1996; Patterson et al. 1998). When looking at *bottlenose dolphin attack* cases, all variables outside of the pathological profile can be considered representative of the animal's condition prior to the physical trauma that was ultimately fatal. In contrast, other acute trauma cases like *grey seal attack* and *bycatch* ellipses overlapped with chronic cause of death profiles, meaning they had shared pathological features. Grey seal attacks often lead to secondary infections, seen here in 100% occurrence of lymph node pathology in this cause of death type, complicating assessments of whether an individual was already compromised or if infection developed post attack (Foster et al. 2019; Davison et al. 2025). The clarity of pathology in bottlenose dolphin attack cases removes such ambiguity, providing clear insight into an animal's health prior to death. It is important to note that there is likely bias in the targeting of harbour porpoises by bottlenose dolphins with a clear preference for animals in *good* nutritional condition. This supports the theory of mass‐dependent predation risk, where bottlenose dolphins selectively target heavier individuals (MacLeod et al. 2007; Barnett et al. 2009). However, these may represent individuals that are otherwise not represented in chronic cause of death and are thus a valuable group to observe for underlying morbidities. Therefore, it can be considered that *bottlenose dolphin attack* cases provide a useful representation of the ‘general’ population's health condition.

Bycatch cases, which have been used as representative of the population in other studies, did not have distinct pathology here (Meager and Sumpton 2016). This may be due to the low sample size in this study, with only 22 bycatch cases making it difficult to characterise a clear cause of death profile. However, there was a 60% occurrence of hepatic lipidosis in bycatch cases, which was the second highest occurrence of this pathological variable across cause of death types. Hepatic lipidosis is typically associated with individuals that have insufficient nutrition and may suggest a bias to individuals in a compromised nutritional state being more susceptible to bycatch (IJsseldijk et al. 2022). However, more extensive study with a greater sample size is needed to further elucidate this finding and its drivers.

### 4.4 Distinct Health Clusters

Hierarchical clustering identified three clusters in the data, representing the most common harbour porpoise profiles, covering a range of health conditions within this population. Cluster 1, the largest group, was mostly made up of individuals in *good* nutritional condition that were *absent* for most pathology and parasite variables and predominantly contained *bottlenose dolphin attack* cases. These animals were in a favourable overall health condition with limited underlying pathology, likely representing the healthy portion of this population. Cluster 1 was dominated by juveniles but also contained the highest number of *neonates*, suggesting that the *juveniles* in this cluster were younger and most similar to neonates, with minimal exposure to stressors. The neonatal class includes individuals aged 0–1 year, juveniles range from 1 to 3 years, and adults from 3 years and older (Ólafsdóttir et al. 2003). This means that some juveniles will be closer in age to neonates, while others approach adult age. Few *adults* were seen in this cluster, but they were still represented here, indicating there is a portion of the adult population in good health with no or limited underlying pathology.

In contrast, cluster 2 was made up of animals in *poor* nutritional condition that were *present* for pathology and had *moderate* to *severe* parasite burdens, indicative of poor health. This cluster was dominated by juveniles and adults and very few neonates, suggesting older juveniles that may be dealing with an onslaught of relatively recently acquired parasites (Lehnert et al. 2014). Parasite burdens were often high in this cluster, suggesting compromised immunity due to the associated chronic‐active immunological response required to deal with high burdens of parasites (Hasegawa et al. 2025). Mortality in this cluster was primarily attributed to *infectious disease* or *starvation/emaciation* cases, both reflecting long‐term health deterioration. This suggests that this cluster represents individuals in the poorest health in this population.

Cluster 3 represented the highest proportion of adults and the lowest proportion of juveniles, with no neonates. The cause of death was dominated by *bottlenose dolphin attack* cases but also had a high number of *infectious disease* cases. Animals in this cluster were in *good* nutritional condition but also *presented* a broad range of pathological features and commonly had *moderate* parasite burdens, highlighting underlying compromised health. This suggests that even nutritionally robust harbour porpoises often have underlying compromised health, which may be missed when looking at BC as an indicator of health (IJsseldijk et al. 2024). This is particularly concerning given the increasing pressures from anthropogenic stressors in marine environments, which may be acting on harbour porpoises already compromised by poor health or underlying pathology.

### 4.5 Pathological Variables and Body Condition Indices

RF models primarily relied on size‐specific variables to predict BC scores in harbour porpoises, with pathological variables contributing less to model performance. Despite the model's high predictive accuracy, its linear structure limits its ability to capture asymptotic trends in BC among larger animals. Moreover, the variables with the greatest influence in the RF suggest that BC does not effectively reflect differences in pathological status. For example, respiratory and hepatic pathology had < 1% influence on the predictive ability of the model despite being identified as important variables for explaining variation in pathology data. Age class and parasitism across various organ systems were the strongest predictors of BCI, both of which directly influence energy reserves by affecting an individual's weight and size (Murphy et al. 2020; Hasegawa et al. 2025). High parasite burdens can have a negative impact on energy balance, as parasites extract nutrients from their host, reducing available resources, and inducing a chronic‐active immunological response that requires a significant compensatory calorie input, consequently lowering BCI (Hasegawa et al. 2025). Similarly, age‐related growth patterns play a crucial role in size, with juveniles and neonates experiencing rapid growth before size plateaus around 3–4 years of age (Murphy et al. 2020). This suggests that BCI alone may not be sufficient to detect subtle variations in health when they do not directly affect energy reserves, such as in cases from cluster 3, and thus gives poor insight into comorbidities and sub‐lethal impacts. Comorbidities are important to consider from a disease and wildlife health perspective, as they allow for the full context of a population's health to be monitored. Future studies should aim to develop a more comprehensive health index that integrates pathological variables, allowing for the maximised use of valuable necropsy data.

In addition to the limitations in BC assessment, our diagnostic categorisation approach (trauma, infection, energetic) also introduces constraints that warrant consideration. The pathological variables used here were grouped into distinct categories to facilitate broad interpretation and comparison of variables, but inevitably involve simplification of complex and potentially overlapping processes. For example, natural ingesta (the ingestion of natural non‐prey material) was classified under ‘energetics’ to reflect the link to compromised nutritional status and energy imbalance. However, it may also reflect pica, which could be attributed to behavioral changes. Similarly, organ pathology was typically categorised as ‘infection’ although in some cases ‘inflammation’ may have been more accurate, particularly where histopathology suggests an immune response rather than a confirmed infection. Such broad categorisation may obscure finer scale distinctions, and future studies using these groupings may wish to utilise detailed histopathological review to refine classifications.

### 4.6 Missing Data and Wider Application

There were a large number of incomplete necropsy reports in the dataset, resulting in over half of the cases being removed during data cleaning stages. This represents a huge loss of information that could have been used to make more robust inferences. Incomplete necropsy reports were most often caused by scavenger damage before examination and carcasses being frozen. Unfortunately, scavenger activity is an unavoidable aspect of the stranding process. Similarly, freezing is generally already avoided where possible due to its known impact on tissue integrity and is only used when no other preservation method is feasible. Imputing missing data offers a solution to this problem, with established causal dependencies providing structure informed by an understanding of the underlying relationships between variables (Ke et al. 2022). This would allow incomplete necropsy reports to be included in analyses, providing a stronger determination of trends and greater statistical power (Piot et al. 2024). Future research should investigate the feasibility of using this approach with pathology data and the potential to utilise imputation to increase sample sizes, allowing for enhanced use of necropsy reports.

## 5 Conclusion

Assessing long‐term population health is an important aspect of wildlife monitoring, as is determining resilience. In this study, we found that a large proportion of adult harbour porpoises in Scotland, regardless of their nutritional condition, had underlying moderate pathologies that suggest compromised health. This indicates that adults in this population may have low capacity to deal with additional stressors, highlighting the need for improving conservation measures. We identified the respiratory and hepatic systems as priority areas for data collection to ensure that representative health assessments can be made and integrated into population‐level analysis. The analytical approach using pathological data in this study simplifies complex and rigorous necropsies and, as such, is suggested as a complement to these practices. By integrating quantified pathological data into simplified, broadscale analysis, more generalised health trends can be revealed. This study emphasises the value of necropsy data in monitoring long‐term health and lays the foundation for future research to refine and expand these approaches. For example, this approach becomes more powerful when used across temporal and spatial scales, establishing long‐term changes in health throughout wider geographical distributions (Dudhat et al. 2022; Albrecht et al. 2024). These methods can also be applied to various wildlife species, supporting health surveillance across different taxa. However, they should be adapted to account for species‐specific biology and regional variations in health baselines and stressors. As environmental pressures on wildlife populations increase, developing efficient, data‐driven methods to assess health will be crucial for informing conservation strategies and mitigating anthropogenic impacts on vulnerable populations.

## Author Contributions

**Rachel L. Lennon:** conceptualization (lead), data curation (lead), formal analysis (lead), investigation (lead), methodology (lead), validation (lead), visualization (lead), writing – original draft (lead). **Jennifer Storm:** data curation (supporting), writing – review and editing (equal). **Rylyn Koger:** data curation (supporting), writing – review and editing (equal). **Elleigh Thompson:** data curation (supporting), writing – review and editing (equal). **Rosie S. Williams:** conceptualization (equal), supervision (equal), writing – review and editing (equal). **Mark P. Dagleish:** conceptualization (equal), methodology (equal), writing – review and editing (equal). **Simon A. Babayan:** supervision (equal), writing – review and editing (equal). **Mariel T. I. ten Doeschate:** data curation (equal), writing – review and editing (equal). **Nicholas J. Davison:** data curation (equal), writing – review and editing (equal). **Andrew C. Brownlow:** conceptualization (equal), data curation (equal), supervision (equal), writing – review and editing (equal).

## Conflicts of Interest

The authors declare no conflicts of interest.

## Supporting information


**Appendix S1:** ece372119‐sup‐0001‐AppendixS1.docx.

## Data Availability

Data is under embargo for a year after publication, after which it can be accessed via Zonodo at this link: https://zenodo.org/records/15393902.
