# Supplementary material for: The Dead Do Tell Tales: Using Pathology Data From Cetacean Necropsy Reports to Gain Insights Into Animal Health
Source: Ecol Evol. 2025 Sep 10;15(9):e72119. doi: 10.1002/ece3.72119 (PMC12422810; doi:10.1002/ece3.72119)
Supplement: Supplementary file 1 — Appendix S1: ece372119‐sup‐0001‐AppendixS1.docx. [file ECE3-15-e72119-s001.docx]

Supplementary Materials

Table S1: Percentage distribution of pathology variable levels across three clusters identified in hierarchical analysis of harbour porpoise pathology data.

| **Variable** | **Level** | **Cluster** | | |
| --- | --- | --- | --- | --- |
|  |  | Cluster 1 | Cluster 2 | Cluster 3 |
| Age class | Adult | 21% | 59% | 65% |
|  | Juvenile | 64% | 39% | 34% |
|  | Neonate | 15% | 2% | 0% |
| Body condition score | Poor | 2% | 10% | 0% |
|  | Moderate | 27% | 55% | 7% |
|  | Good | 59% | 35% | 72% |
|  | Very Good | 12% | 0% | 21% |
| Cause of death * | Bottlenose dolphin attack | 29% | 4% | 51% |
|  | Infectious disease | 15% | 42% | 24% |
|  | Starvation/emaciation | 12% | 17% | 0% |
| Respiratory pathology | Absent | 73% | 28% | 0% |
|  | Present | 27% | 72% | 100% |
| Hepatic pathology | Absent | 70% | 20% | 17% |
|  | Present | 30% | 80% | 83% |
| Respiratory parasites | Absent | 50% | 7% | 3% |
|  | Mild | 29% | 26% | 28% |
|  | Moderate | 6% | 33% | 48% |
|  | Severe | 15% | 33% | 21% |
| Hepatic parasites | Absent | 80% | 41% | 28% |
|  | Mild | 12% | 17% | 38% |
|  | Moderate | 8% | 29% | 31% |
|  | Severe | 0% | 13% | 3% |

*Incomplete cause of death, with only most prevalent three levels included in this table.

**
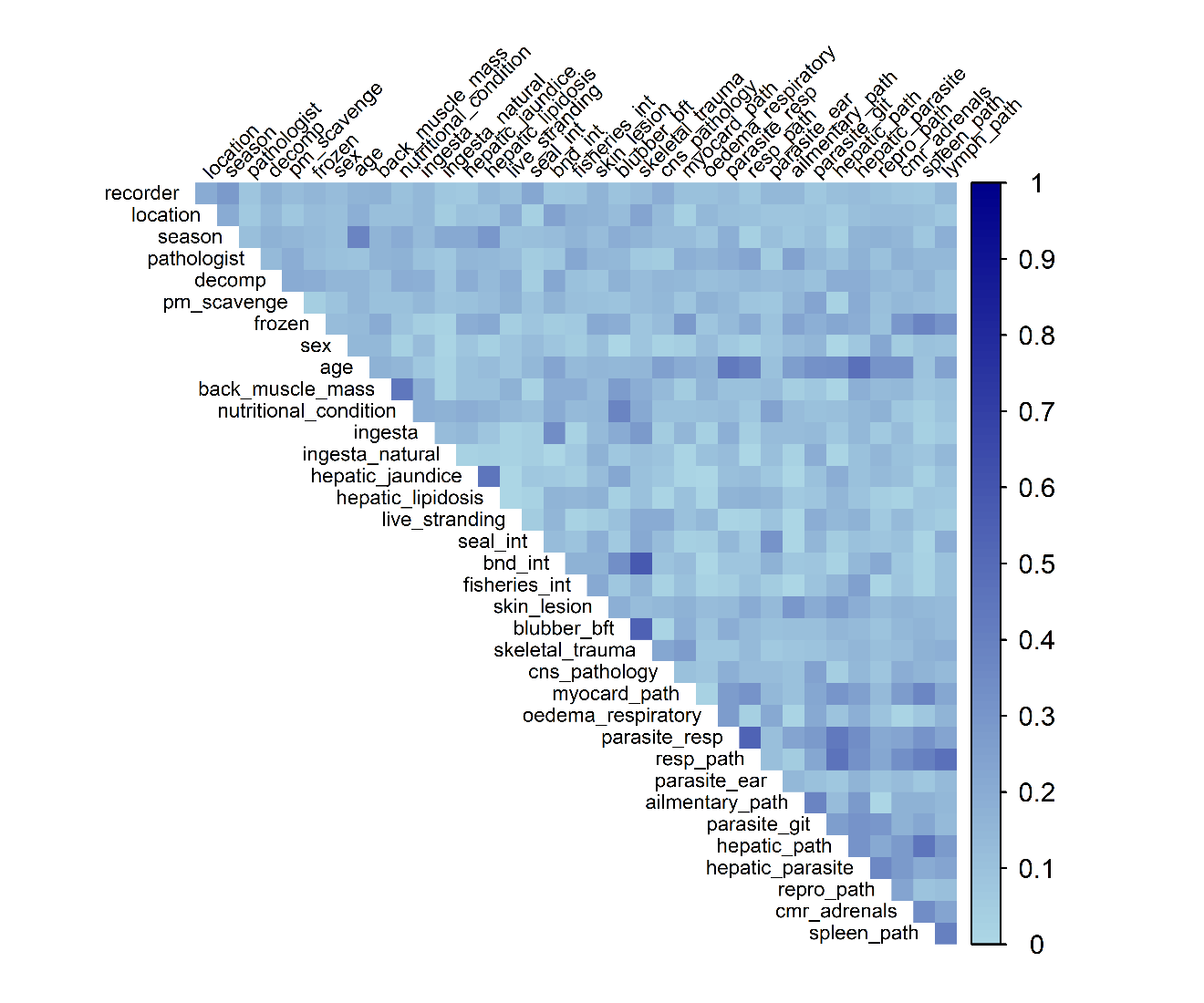
**

Figure S1: Cramer’s V correlation coefficient matrix between pathological variables. Values closer to 1 (dark blue), have a higher association than values closer to 0 (light blue). Values that are >0.5 have a very strong association.


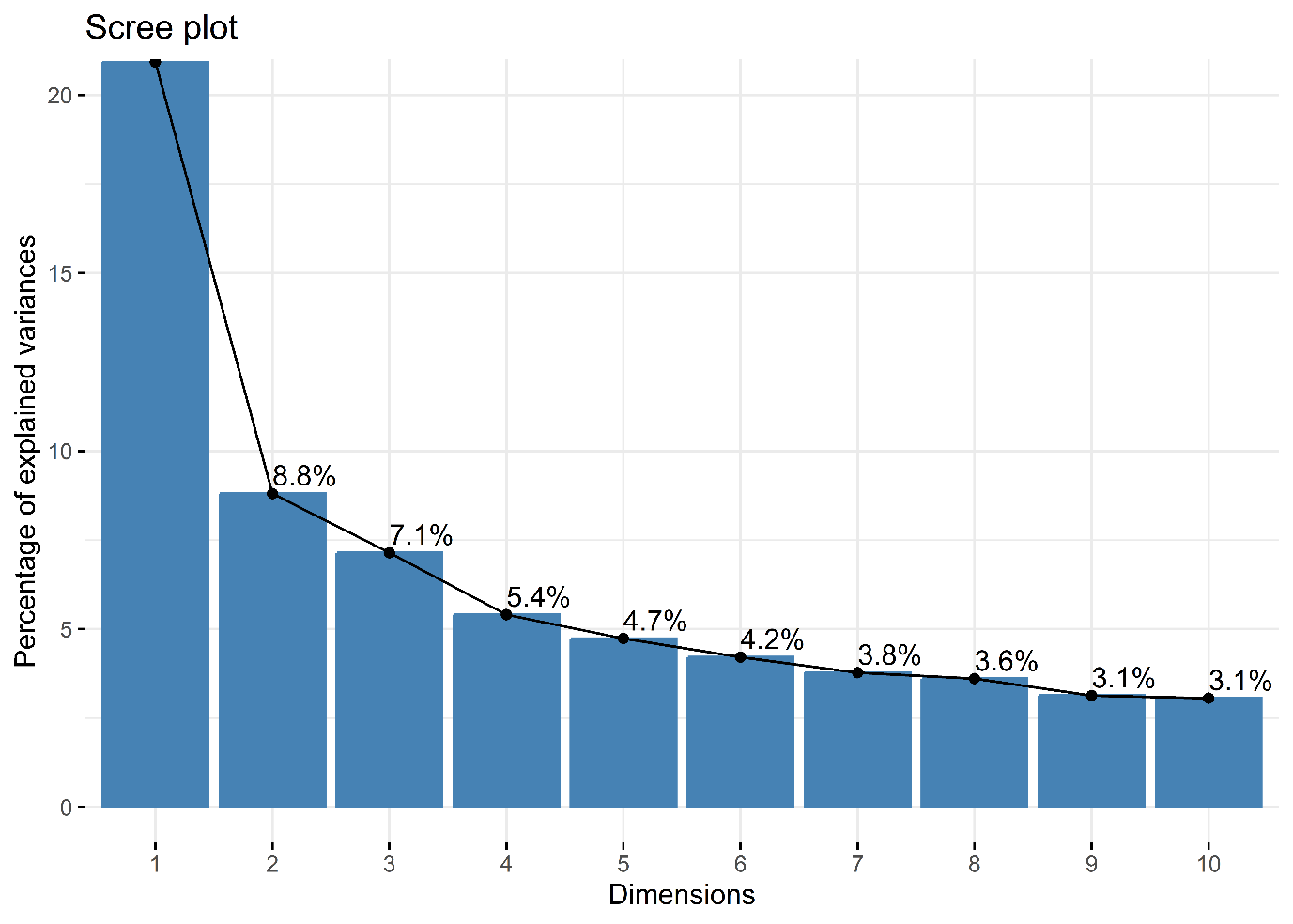


Figure S2: Scree plot illustrating the percentage of variance explained by the first 10 dimensions of the Multiple Correspondence Analysis.


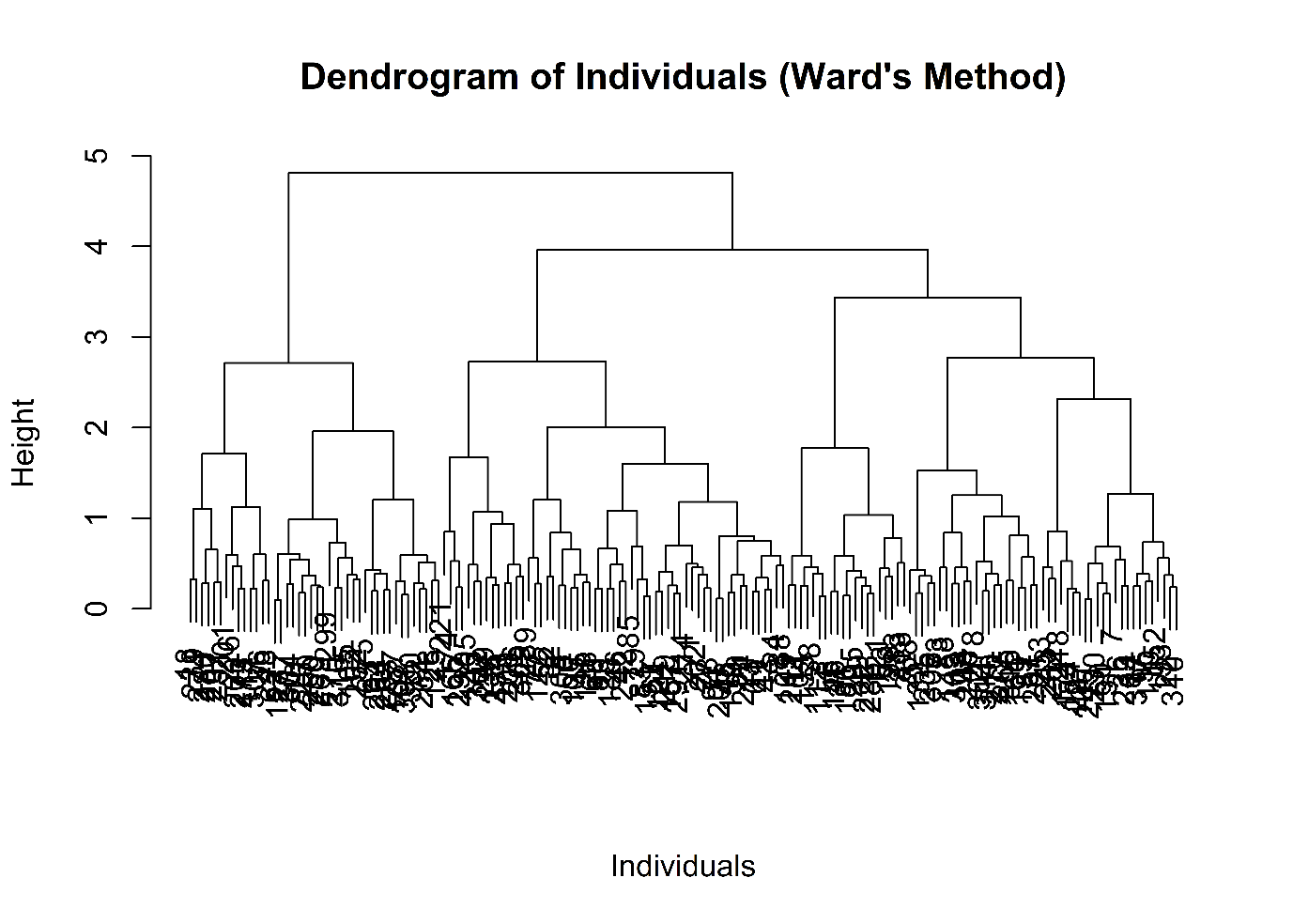


Figure S3: Dendrogram of hierarchical clusters of stranded individuals based on Multiple Correspondence Analysis results.
